# Different patterns of short-term memory deficit in Alzheimer's disease, Parkinson's disease and subjective cognitive impairment

**DOI:** 10.1016/j.cortex.2020.06.016

**Published:** 2020-11

**Authors:** Nahid Zokaei, Annie Sillence, Annika Kienast, Daniel Drew, Olivia Plant, Ellie Slavkova, Sanjay G. Manohar, Masud Husain

**Affiliations:** a Oxford Centre for Human Brain Activity, Wellcome Centre for Integrative Neuroimaging, Department of Psychiatry, University of Oxford, Oxford, OX3 7JX, UK; b Department of Experimental Psychology, University of Oxford, Oxford, OX1 3UD, UK; c Oxford NIHR Biomedical Research Centre, UK; d Wellcome Centre for Integrative Neuroimaging, John Radcliffe Hospital, Oxford, UK; e Nuffield Department of Clinical Neurosciences, University of Oxford, Oxford, OX3 9DU, UK

**Keywords:** Short-term memory, Binding, Alzheimer's disease, Parkinson's disease

## Abstract

It has recently been proposed that short-term memory (STM) binding deficits might be an important feature of Alzheimer's disease (AD), providing a potential avenue for earlier detection of this disorder. By contrast, work in Parkinson's disease (PD), using different tasks, has suggested that the STM impairment in this condition is characterised by increased random guessing, possibly due to fluctuating attention. In the present study, to establish whether a misbinding impairment is present in sporadic late-onset AD (LOAD) and increased guessing is a feature of PD, we compared the performance of these patient groups to two control populations: healthy age-matched controls and individuals with subjective cognitive impairment (SCI) with comparable recruitment history as patients. All participants performed a sensitive task of STM that required high resolution retention of object-location bindings. This paradigm also enabled us to explore the underlying sources of error contributing to impaired STM in patients with LOAD and PD using computational modelling of response error.

Patients with LOAD performed significantly worse than other groups on this task. Importantly their impaired memory was associated with increased misbinding errors. This was in contrast to patients with PD who made significantly more guessing responses. These findings therefore provide additional support for the presence of two doubly dissociable signatures of STM deficit in AD and PD, with binding impairment in AD and increased random guessing characterising the STM deficit in PD. The task used to measure memory precision here provides an easy-to-administer assessment of STM that is sensitive to the different types of deficit in AD and PD and hence has the potential to inform clinical practice.

## 1 Introduction

With ~45% of individuals aged >85 years being diagnosed with Alzheimer's disease (AD) (Liu, Liu, Kanekiyo, Xu, & Bu, 2013), one of the key priorities of healthcare has become the identification of individuals using sensitive measures that can be administered relatively rapidly. Cognitive deficits, specifically memory-related impairments, are an important feature of AD. Although much of the focus previously has been on long-term memory (LTM) or episodic memory, recent investigations have shown that patients with either familial AD (FAD) or late-onset AD (LOAD) can also have significant deficits in short-term memory (STM) (Guazzo, Allen, Baddeley, & Sala, 2020; Liang et al., 2016; Parra et al., 2009, 2010, 2011, 2015).

These findings intersect with recent models of memory which propose that the medial temporal lobes (MTL) – and specifically the hippocampus, a region often implicated relatively early in AD–is involved not only in LTM but also plays a role in STM. According to this perspective the hippocampus might play a role in a specific computation: retention of high resolution binding of features belonging to a memory episode, regardless of retention duration, short or long (Olson, Page, Moore, Chatterjee, & Verfaellie, 2006; Pertzov et al., 2013; Yonelinas, 2013). Consistent with this proposal, several studies have now provided evidence for binding deficits in STM in individuals with focal MTL lesions as well as those with AD (Della Sala, Parra, Fabi, Luzzi, & Abrahams, 2012; Koen, Borders, Petzold, & Yonelinas, 2016; Liang et al., 2016; Parra et al., 2010, 2011, 2009; Pertzov et al., 2013; Zokaei, Nour, et al., 2018).

A series of pioneering investigations that have provided evidence for binding impairments in patients with AD by Parra and colleagues (Della Sala et al., 2012; Guazzo et al., 2020; Parra et al., 2009, 2010, 2011, 2015) used variants of a change-detection task in which LOAD or FAD cases were presented with memory arrays consisting of either single features (e.g., colours), or multiple features bound together in a single object (e.g., coloured objects). Participants were asked to keep these in mind and later, following a brief delay, detect any changes in a second array compared to the one held in memory. Individuals with AD consistently performed worse in the binding conditions only (Della Sala et al., 2012; Guazzo et al., 2020; Kozlova, Parra, Titova, Gantman, & Sala, 2020; Parra et al., 2009, 2010, 2011, 2015).

The change-detection studies described above employed a paradigm in which participants make either correct or incorrect (binary) responses. Performance on the task can be used to estimate the number of items which people can recall correctly from STM (Luck & Vogel, 1997). However, simply because an individual fails to recall an item does not mean that all the information regarding that item was completely lost from memory. In other words, change detection tasks do not provide a measure of the quality of memory representations when an observer makes an incorrect response. Moreover, the condition of interest in AD, the binding condition, required an additional operation compared to single-feature trials (Della Sala et al., 2012; Guazzo et al., 2020; Parra et al., 2009, 2010, 2011, 2015). Thus, participants had to remember both single features as well as their associations with one another, hence potentially limiting any direct comparisons made with trials in which only single features were to be retained.

A recent theoretical and empirical approach to STM employs a different means to probe STM. It allows researchers to examine the resolution with which items are retained in memory by asking participants to respond using a continuous, rather than binary, response (for a review see: Ma, Husain, & Bays, 2014; Fallon, Zokaei, & Husain, 2016), thereby addressing some of the limitations of change detection methods raised above. In these continuous reproduction tasks, participants are required to reproduce the exact quality of remembered features in an analogue response space which provides a more sensitive measure of STM (P.M. Bays, Catalao, & Husain, 2009; Gorgoraptis, Catalao, Bays, & Husain, 2011; Pertzov, Dong, Peich, & Husain, 2012; Zokaei, Gorgoraptis, Bahrami, Bays, & Husain, 2011). One such paradigm which has also been validated in patients with focal MTL lesions (Pertzov et al., 2013; Zokaei, Nour, et al., 2018) and in patients with FAD (Liang et al., 2016) examines the resolution with which object-location bindings are retained in STM. The results showed that FAD and MTL lesion cases do indeed have deficits in feature binding (Liang et al., 2016; Pertzov et al., 2013; Zokaei, Nour, et al., 2018), supporting previous studies using change detection tasks in FAD (Parra et al., 2010). However, this task has not yet been tested in sporadic LOAD cases.

Continuous reproduction STM paradigms that measure recall precision can also provide a means to dissect out sources of error contributing to the pattern of performance using modern analytical techniques (P.M. Bays et al., 2009; Grogan et al., 2019). Specifically, three different contributions to impaired performance can be separated using these methods: error due to imprecision (noisiness) of recall, increased misbinding (or swap) errors in which participants report a feature associated with another item in memory, or alternatively increased proportion of random guesses. For example, in an object-location binding task, a swap occurs when participants report the location of another item in memory and hence misbinding the objects and their corresponding locations. Therefore, without needing to separate trial-types depending on the type of information that is retained (single features vs. bound objects), it is possible to isolate the underlying associated impairment in STM: whether the errors are driven largely by imprecision (noisiness) of recall, random guessing or misbinding (swaps).

This dissection of the nature of errors contributing to STM impairments is important because it has the potential to provide mechanistic insights into the cognitive processes that are dysfunctional in a brain disorder. It is now known that several different neurodegenerative conditions can lead to STM deficits (e.g., Panegyres, 2004) but the underlying mechanisms might be different across different diseases. For example, patients with Parkinson's disease (PD) have long been known to exhibit STM impairments, apparent at the very earliest stages of the disease (e.g., Dujardin, Degreef, Rogelet, Defebvre, & Destee, 1999; Muslimovic et al., 2005; Owen et al., 1992; 1993; 1997; Verbaan et al., 2007). In contrast to work in AD, research on STM deficits in PD using a different type of continuous response paradigm (which examined colour-orientation binding) has shown that these individuals and those at risk of developing PD make significantly more random guessing responses than healthy controls (Rolinski et al., 2015; Zokaei, McNeill, et al., 2014). Thus, the mechanism underlying the STM deficit in PD might be distinct to that observed with patients with hippocampal deficits such as patients with AD.

To the best of our knowledge, however, LOAD and PD cases have not previously been compared directly using a continuous reproduction task, although other researchers have compared LOAD cases to PD dementia using a change detection task (Della Sala et al., 2012). This study reported increased misbinding in LOAD but no visual STM deficit in PD patients who have developed dementia. A subsequent investigation by the same group compared LOAD patients to PD cases with or without dementia (Kozlova et al., 2020). The authors concluded again that, although misbinding is increased in LOAD, the PD cases–either with or without dementia–show no significant visual STM deficit compared to healthy controls on change detection performance. It remains to be established therefore if LOAD and PD cases have doubly dissociable patterns of underlying STM deficit–with increased misbinding in AD and increased guessing in PD–using the same reproduction task to test both groups.

To put this hypothesis to the strongest test, it would be important to compare LOAD cases with PD patients without dementia because it is now known that mean onset to dementia is 10 years after the diagnosis of PD (Aarsland & Kurz, 2010). If it is possible to demonstrate, on the same task, an underlying cause of impaired STM performance in PD cases without dementia that is doubly dissociable from that in LOAD, at comparable times since diagnosis, that would potentially provide strong evidence for distinctly different cognitive mechanisms contributing to STM dysfunction in the two diseases. In this study, therefore, we examined visual STM performance and the sources of error in LOAD and PD cases without dementia, who were not significantly different from each other in terms of diagnosis duration, on the same continuous reproduction task.

In addition, we examined performance of two control groups. First, we studied individuals with subjective cognitive impairment (SCI). These patients were included as they present to clinics complaining of everyday memory difficulties, but are not diagnosed with any neurological disorder at the time of testing (Stewart, 2012). They therefore provide a potentially important second comparison group as their subjective experience of their memory abilities is impaired, as is often the case in AD, but they do not have objective evidence of a significant neurodegenerative condition. Therefore, we would not expect *most* SCI patients to show a visual STM deficit characterized by misbinding as we would in AD, despite the fact that both groups of patients might complain of memory deficits. The definition of SCI we use here is different to authors Jessen et al. (2014) who specifically wish to develop criteria for individuals with subjective cognitive decline (SCD) who are in the pre-clinical phase of AD, prior to mild cognitive impairment (MCI). Our definition is the wider one of all patients who report difficulties with their memory but do not have evidence of significant objective deficits and are not given a diagnosis of a neurodegenerative disorder (Howard, 2020). Lastly, in addition to SCI cases, we also examined a group of healthy controls without significant memory complaints, as they provide a second control or baseline of performance.

In the present study, we used an object-location continuous reproduction binding task to examine STM performance (Pertzov et al., 2013; Zokaei et al., 2017; Zokaei, Čepukaitytė, et al., 2018) across all four groups of individuals: LOAD, PD, SCI and healthy controls. The task required participants to report the exact location of remembered objects and, importantly in addition, enabled us to explore the underlying sources of error contributing to impaired STM using computational modelling of response error. The paradigm was developed for clinical use following a series of studies in healthy people challenged the view that the best way to characterize STM might be in terms of the number of items it can hold (P.M. Bays & Husain, 2008; Ma et al., 2014; Wilken & Ma, 2004). Instead, data from several investigations have demonstrated that the use of continuous (rather than discrete) error measures provides a view of STM that is far more flexible than previously envisaged. Moreover, these tasks–sometimes referred to as precision STM tasks–readily permit modelling of the sources of error contributing to memory performance (P.M. Bays et al., 2009; Ma et al., 2014).

## 2 Methods

### 2.1 Participants

No part of the study procedure was pre-registered prior to the research being conducted. Overall, eighty-nine individuals participated in this study. This included:

• 20 patients with a diagnosis of LOAD based on the NIA-AA core clinical criteria for probable AD (McKhann et al., 2011)**,** 13 of whom were on donepezil
• 20 patients with a diagnosis of PD based on the UK Parkinson's Society Brain Bank criteria (Hughes, Daniel, Kilford, & Lees, 1992) (mean daily levodopa equivalent dose = 658 mg)
• 24 people with SCI defined as people who presented with complaints about their memory but clinically did not present with symptoms of MCI or dementia (Howard, 2020) on the basis of the history obtained from the patient and an informant, and on the basis of performance on the Addenbrookes Cognitive Examination-III (ACE-III). Of the 24 participants with SCI, 6 had anxiety (one on anxiety medication), 6 had depression (two were on antidepressants) and lastly 3 reported poor sleep (though none were on any specific medication for this) and one of these cases also reported anxiety.
• 25 healthy controls (HCs).

Patients were recruited over three years through a neurology clinic with a specialist interest in cognitive disorders at the John Radcliffe Hospital, Oxford and were tested on one occasion. Control participants were recruited from Oxford Dementia and Ageing Research database.

Demographics, patient information and details of statistical comparisons are presented in Table 1. There was no significant difference in age of patients with AD, PD, SCI and HCs. The Addenbrooke's Cognitive Examination (ACE III) test was administered as a general cognitive screening test to patients with AD, PD, SCI and HCs. Patients with AD scored significantly lower on the ACE compared to healthy controls, patients with PD and individuals with SCI (all Bonferroni corrected *p* < .001). There was no significant difference in ACE scores between the PD, SCI and healthy controls. On average, PD cases had been diagnosed slightly longer than AD patients but this difference was not significant.

**Table 1:** Demographics characteristic of patients and healthy control participants.

	N	Gender (f/m)	Age	ACE score	Disease duration
**HC**	25	14/11	67.4 (6)	96 (3.2)	–
**AD**	20	9/11	68 (7.0)	75.6 (8.5)	3.9 (2.2)
**PD**	20	9/11	64 (6)	93.8 (4.6)	4.6 (3.1)
**SCI**	24	13/11	67 (10)	92 (5)	–
**p**	–	n.s	n.s	<.001^a^	n.s.

Means and SDs in parentheses.

a One-way ANOVA performed on the ACE scores demonstrated a significant difference. Post-hoc Tukey test showed that AD patients obtained significantly lower scores than healthy controls, than PD patients and compared to SCI cases (all *p* < .001). Chi-square tests showed no significant difference in gender proportion between any of the groups (*p* > .46 for all comparisons). One-way ANOVA for age showed no significant difference for age (*p* > .60). For disease duration, unpaired *t*-tests showed no significant difference between AD and PD cases (*t* (38) = 1.08, *p* = .29).

An approximation of the sample size was determined based on previous studies on short-term memory performance, using a similar task to the one employed here, in various patient groups and individuals at risk of developing neurodegenerative disorders (Liang et al., 2016; Rolinski et al., 2015; Zokaei, McNeill, et al., 2014; Zokaei et al., 2017; Zokaei, Nour, et al., 2018; Zokaei, Čepukaitytė, et al., 2018).

All participants had normal or corrected to normal vision and HC participants and individuals with SCI had no neurological disorders at time of testing. The study was approved by the local NHS ethics committee and all participants provided fully informed consent to task procedure.

### 2.2 Short-term memory task

**Fig. 1:**
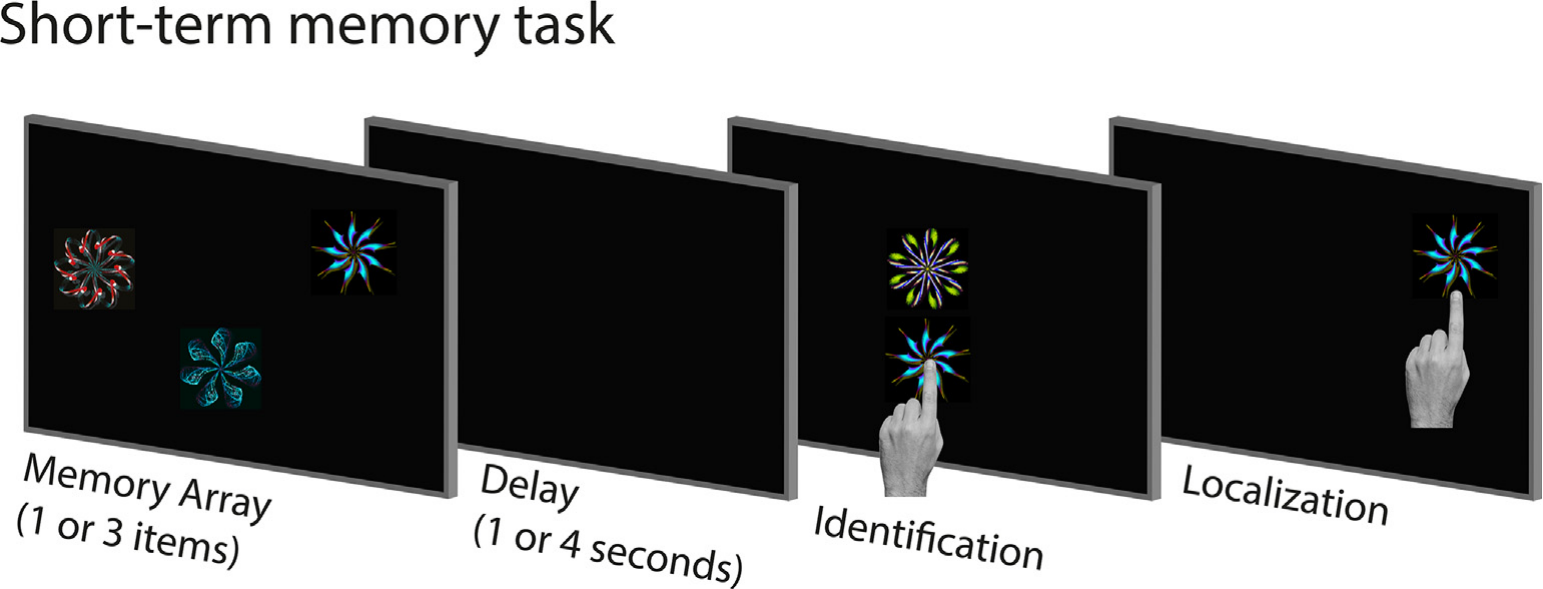
**Short-term memory task.** Schematic of the short-term memory task. Participants were presented with a memory array followed by a delay. They were then presented with two fractals, one from the memory array and a foil. On a touchscreen computer, participants first had to touch the fractal they had seen before (in the memory array) and drag it its remembered location.

The STM task was identical to one previously used (Zokaei et al., 2017; Zokaei, Nour, et al., 2018) (Fig. 1). It was presented on a touchscreen (Inspiron All-in-One 2320; DELL) with a 1920 × 1080 pixel resolution (corresponding to 62 × 35 of visual angle) at a viewing distance of approximately 62 cm.

In brief, in each trial, participants were presented with 1 or 3 abstract images (fractals) comprising the memory array, for 1 or 3 s. The memory array was then followed by either 1 or 4 s of a black screen, before recall when participants were presented with 2 fractals on the vertical meridian at screen centre. One of the fractals had appeared in the preceding memory array while the other was a foil, i.e., a novel fractal. Participants were asked to select the fractal that had appeared in the memory array by touching it (identification accuracy). Once one of the fractals was selected, participants had to drag it on the touchscreen it to its remembered location (localization memory), and confirm their response with a key press. The localization phase of the task provides a continuous measure of error, rather a binary correct/incorrect response.

Stimuli were randomly selected from a pool of 60 coloured fractals, with a maximum height and width of 120 pixels (4°of visual angle). The location of each fractal was random, but with a minimum distance of 3.9° from the monitor edge, and a minimum distance of 6.5° from screen centre.

Participants completed between 2 and 4 blocks of the task, depending on availability. Each block consisted of 16 trials in which 1 item was presented in the memory array (8 per delay duration) and 32 trials in which 3 items had to be retained in memory (16 per delay duration). The full task took approximately 30 min to complete. Participants were familiarized with task procedure prior to the testing by completing 8 practice trials with increasing difficulty.

### 2.3 Analysis

#### 2.3.1 Behavioural analysis

Identification accuracy and localization error were used as an overall measure of performance. Identification accuracy is calculated as the proportion of trials in which participants correctly select the item that was previously in the memory array. Trials in which the correct item was not identified were excluded from subsequent analysis. Localization error was then calculated as the difference between the location of the item in the memory array and the reported location in pixels.

#### 2.3.2 Mixture modelling of error

STM precision tasks, such as the one employed here, also provide a means to dissect out sources of error contributing to the pattern of performance (P.M. Bays et al., 2009; Gorgoraptis et al., 2011; Ma et al., 2014). In these paradigms, error can potentially arise from three distinct sources. First, error can be due to variability in memory for the probed item (Imprecision). In other words, how well a feature, here location, is stored in memory. Second, participants may make random errors, because on some trials, they may be simply guessing (Guesses). Lastly, error can arise from misreporting features of the non-probed (other) items that were presented in the memory array (Swaps). In such cases, participants’ responses might be systematically biased by other items that were encoded into STM. This general model has successfully been applied previously to one dimensional features in memory such as motion, orientation or colour in both healthy population (e.g., P.M. Bays et al., 2009; Gorgoraptis et al., 2011; Zokaei et al., 2011) as well as the ageing population and patients with various neurological disorders (Mok, Myers, Wallis, & Nobre, 2016; Peich, Husain, & Bays, 2013; Rolinski et al., 2015; Zokaei, McNeill, et al., 2014).

Here, to identify sources of error contributing to overall STM performance, a specific model for this type of task was applied to localization error data for set size three trials (Grogan et al., 2019). According to this model, as in previous applications to other stimuli noted above, error can arise due to increased imprecision (Fig. 3a left panel), random responses due to guesses (Fig. 3a middle panel), or swap/misbinding errors (Fig. 3a right panel). In this case, imprecision refers specifically to the noisiness (variability) of response around the true location of the probed item which had appeared in the memory display. Random guessing responses are those that are classed as occurring at locations other than the probed item or any of the other items that had been in the memory display. Finally, swap (misbinding) errors are those in which the responses fall in the locations of items that had been in the memory display but were not actually probed. Thus, swap errors arise in trials in which participants pick the correct fractal but place it in the location of one of the other (non-probed) items from the memory array.

The model is described by the following equation:

$$\text{P }\left(\hat{\theta }\;\right)=\text{α}\psi_{\kappa }\left(\hat{\theta }\;-\theta \right)+\;\beta \frac{1}{M}\sum_{i}^{m}\psi_{\kappa }\left(\hat{\theta }\;-\;\phi_{i}\right)+\;\gamma \frac{1}{A}$$

where the free parameters of α, β, γ, and κ, correspond to proportion of target responses, swaps, guesses and the imprecision respectively. Moreover, $\hat{\theta }$ parameter corresponds to the response, θ to the target, $\phi_{i}$ to the non-probed item's coordinates, and ψ to the bivariate Gaussian distribution, and *A* to the screen dimensions.

In this model, swaps are assumed to be similar to target responses, except that they are centred on the locations of non-probed items. Thus, they take the form of a multivariate Gaussian distribution with the same imprecision parameter as the probed target item. Guesses, however, are assumed to be entirely unrelated to any stimuli locations and take the form of a uniform distribution across the entire screen. This, therefore, reflects a random guess similar to what would happen if the participant had either entirely forgotten all the stimuli, or effectively had their eyes shut during stimulus presentation. Put simply, responses close to non-probed items are more likely classed as swaps (depending on the imprecision parameter), while responses far away from both probed and non-probed items (hence all items in memory) are more likely classed as guesses.

Separate mixed ANOVAs were used, with set size and delay as within-subject factors, and participant group as between-subject factors. For non-normally distributed data, appropriate transformation was applied to meet the requirements of ANOVA. An estimate of effect size is reported as eta-squared (reported for significant effects).

We report how we determined our sample size, all data exclusions (if any), all inclusion/exclusion criteria, whether inclusion/exclusion criteria were established prior to data analysis, all manipulations, and all measures in the study.

Legal restrictions that are beyond our control prevent us from publicly archiving the task and analysis scripts used in this research. Specifically, for commercial use, these can be obtained through licensing agreement with Oxford University Innovation Ltd. These digital materials will, however, be shared freely on request with research groups and non-profit making organisations provided they agree in writing not to share them with commercial parties or use them for profit.

The conditions of our ethics approval do not permit public archiving of the data supporting this study. Readers seeking access to this data should contact the lead author, Prof Masud Husain. Access will be granted to named individuals in accordance with ethical procedures governing the reuse of sensitive data. Specifically, to obtain the data, requestors must complete a formal data sharing agreement, including conditions for secure storage of sensitive data.

## 3 Results

Due to participant availability, a few patients and healthy controls did not complete sufficient trials to examine the effect of memory delay on performance. Hence, for the purposes of this analysis performance across the two retention delays (1 or 4 s) was collapsed to allow for the investigation of the impact of memory set size on performance. All post-hoc *t*-tests were Bonferroni corrected.

### 3.1 Behavioural performance

**Fig. 2:**
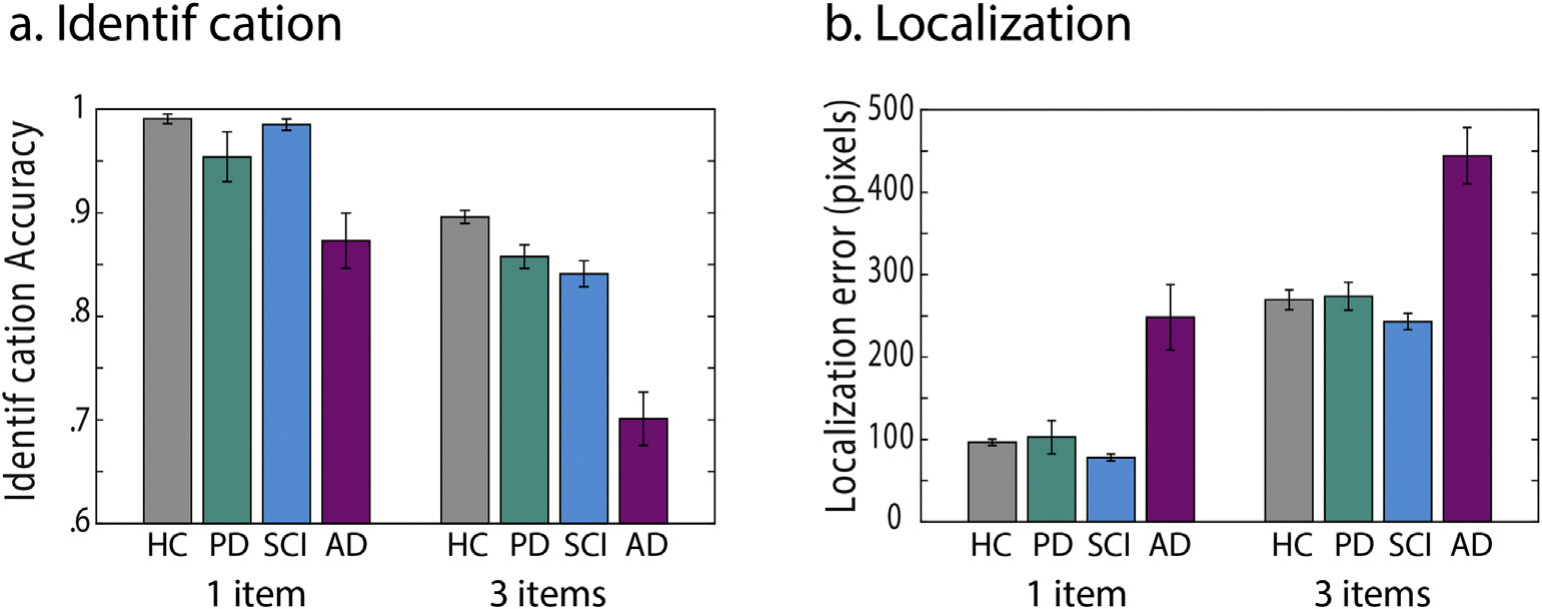
Short-term memory performance. Behavioural task performance, for identification accuracy (a) and localization error (b) for 1 and 3 item conditions for patients with AD, PD, SCI and healthy controls (HC).

For identification accuracy, that is proportion of trials in which participants correctly identified the fractal, a repeated measures ANOVA was performed with set size as a within-group factor (1 or 3 items) and group as between-subject factor. There was a significant effect of set size on identification accuracy (*F* (1,85) = 191, *p* < .001, η^2^_p_ = .69, Fig. 2a Identification) with reduced identification accuracy when 3 items had to be remembered compared to when only 1 had to be retained. In addition, there was a significant main effect of group (*F* (3,85) = 24.4, *p* < .001, η^2^_p_ = .46) and a significant interaction between set size and group (*F* (3,85) = 4.17, *p* = .008, η^2^_p_ = .13), indicating that memory load affected the groups differently.

**Fig. 3:**
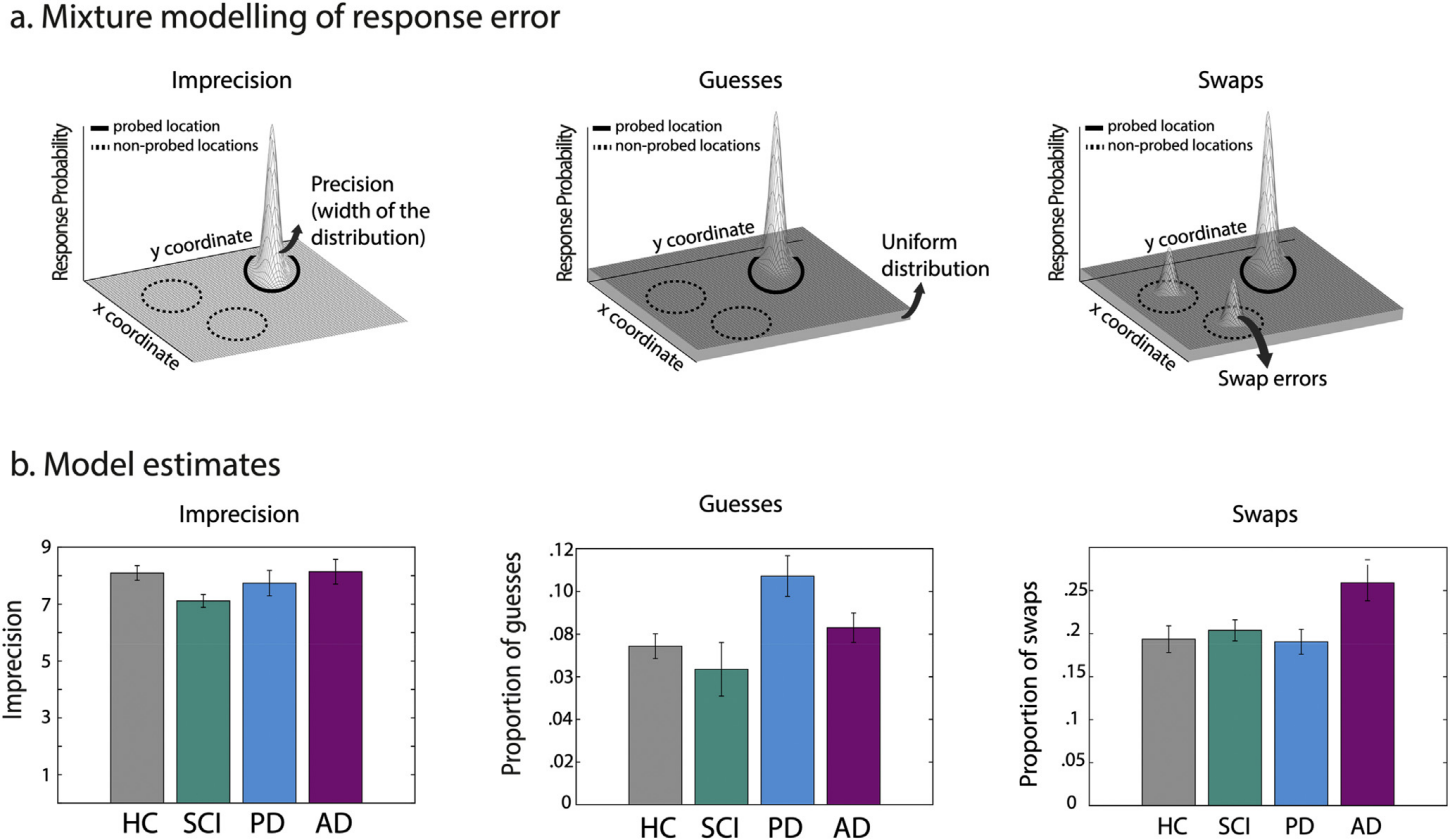
**Computational modelling of response error in STM**. a) Error can arise due to localization imprecision, captured by changes in the gaussian distribution centred on the probed location (right panel), proportion of guesses, captured a uniform distribution (middle panel) or proportion of swap (binding) errors, captured by gaussian distributions centred on the non-probed memory items (right panel). b). Model estimates in different groups of participants demonstrate that patients with PD show increased proportion of guesses while patients with AD make significantly more swap (binding) errors.

This interaction was followed up with two one-way ANOVAs per memory set size. For set size 1, there was a significant effect of group on performance (*F* (3,85) = 8.7, *p* < .001, η^2^_p_ = .24). Bonferroni corrected post-hoc tests revealed significant difference in performance between AD patients compared to HC participants (*p* < .001), patients with PD (*p* < .001) and individuals with SCI (*p* < .001). For set size 3, there was also a significant effect of group (*F* (3,85) = 32.4, *p* < .001, η^2^_p_ = .53), with AD patients performing significantly worse than HCs, individuals with SCI and PD (all *p* < .001), in Bonferroni corrected post-hoc comparisons. Patients with PD did not perform significantly different compared to HCs and individuals with SCI.

We next examined localization memory by measuring the distance between the reported and true location of the probed item. There was a significant main effect of set size (*F* (1,85) = 672, *p* < .001, η^2^_p_ = .9), with larger localization error in trials with 3 compared to 1 fractal and a significant main effect of group (*F* (3,85) = 18, *p* < .001, η^2^_p_ = .39). Post-hoc, Bonferroni corrected comparisons revealed AD patients had significantly greater localization error compared to HC, PD and SCI groups (all *p* < .001) (Fig. 2b localization). Patients with PD did not perform significantly different compared to HCs and individuals with SCI.

### 3.2 Mixture modelling of error

Application of mixture modelling to data from STM precision tasks, such as the one employed here, provides a means to dissect out sources of error contributing to the pattern of performance (P.M. Bays et al., 2009; Gorgoraptis et al., 2011; Ma et al., 2014). A recent additional analytical technique for the type of task used here (Grogan et al., 2019) allowed us to estimate the proportion of responses arising from three sources of error:

• Imprecision of response around the true location of the correctly identified item (Fig. 3a left panel)
• Random responses due to guesses (Fig. 3a middle panel) where the correctly identified item was dragged to a location which was neither its true location nor the location of the other two (non-probed) items that had appeared in the memory display
• Swap or misbinding errors (Fig. 3a right panel) where participants select the correct fractal at probe but place it in the location of one of the other two (non-probed) items from the memory array.

The parameters returned from the model reflect the proportion of responses classed as each type of error (see Table 2 for means and standard deviations of model estimates per participant group).

**Table 2:** Means and standard deviations for model estimates of imprecision, guesses and swaps.

	Imprecision *Set Size 1*	*Set Size 2*	Guesses *Set Size 1*	*Set Size 2*	Swaps *Set Size 2*
***HC***	5.8 1.07	8.1 1.3	.066 .022	.074 0.029	.19 .078
***AD***	6.5 1.8	8.1 1.9	.063 .02	.083 .03	.26 .09
***PD***	5.5 2.4	7.7 1.9	.062 .022	.11 .042	.19 .064
***SCI***	5.8 2.6	7.1 1.1	.062 .025	.043 .02	0.2 .06

For model estimates of imprecision, proportion of random responses and proportion of swaps, a repeated measures ANOVA was performed with group as between-subject factor. There was no significant effect of group on model estimate of imprecision (*F* (3,85) = 2.09, *p* = .108, η^2^_p_ = .069, Fig. 3b–**Imprecision**). For model estimate of proportion of guesses however, there was a significant main effect of group (*F* (3,85) = 3.94, *p* = .011, η^2^_p_ = .122, Fig. 3b–**Guesses**). Post-hoc, Bonferroni corrected comparisons revealed that patients with PD made significantly more guesses compared to both HC (*p* = .013) and SCI participants (*p* = .001). AD patients did not make significantly more guesses compared to HCs and SCI participants.

Lastly, we examined the effect of group on proportion of swap (binding) errors using a one-way ANOVA with group as between-subject factor. There was a significant main effect of group (*F* (3,85) = 3.79, *p* = .013, η^2^_p_ = .12, Fig. 3b–**Swaps**) and Bonferroni corrected post-hoc comparisons revealed that patients with AD made significantly more swaps compared to HC (*p* = .004) and SCI participants (*p* = .017) as well as patients with PD (*p* = .005).

## 4 Discussion

In the present study we examined STM performance in patients with LOAD versus PD, SCI and healthy controls using a sensitive, continuous analogue reproduction task that measures the retention of bound object-locations (Fig. 1). In line with previous research, we found a selective impairment of feature binding in the STM performance of patients with LOAD compared to all other tested groups (Fig. 3b). A previous study in FAD cases also demonstrated increased misbinding in asymptomatic cases, prior to the onset of dementia (Liang et al., 2016). Increased binding errors in patients with LOAD is also consistent with the results of a series of previous studies, using a different (change-detection) methodology, which demonstrated higher rates of misbinding in patients with LOAD and FAD (Della Sala et al., 2012; Guazzo et al., 2020; Parra et al., 2009, 2010, 2011, 2015). Together, these findings provide growing support for the view that AD is associated not only with LTM but also STM impairments, and that increased misbinding might be an important signature of STM deficits in the condition.

Classically, it has been proposed that the MTL and hippocampus in particular play a key role in retention of relational binding of features belonging to an episode in LTM (e.g., Davachi, 2006). However, deficits of STM retention of object-location bindings as demonstrated here and by change-detection studies in patients with LOAD, who typically have MTL atrophy, points to a general role of the MTL that extends beyond the traditional distinction between long vs. short-term memories. In fact, it highlights a computation that might be shared between STM and LTM, namely the high-resolution binding of features to perceive and maintain coherent and bound objects (Yonelinas, 2013). Complementary to this view, precise retention of object-locations even for short durations has been found to rely on MTL structures (Koen et al., 2016; Liang et al., 2016; Libby, Hannula, & Ranganath, 2014; Pertzov et al., 2013; Zokaei, Nour, et al., 2018).

Although the results of these studies point to a key role of the MTL–across different pathologies–in feature binding in visual STM, in the context of neurodegenerative disorders it would be important to consider whether binding deficits can distinguish AD from other conditions that are either associated with neurodegeneration or memory complaints. To this end, in this study we compared LOAD cases to three groups: PD patients with diagnosis duration that is not significantly different to the AD cases; people with SCI who present with subjective memory complaints but are not considered to have AD after investigation; and healthy controls.

Previously other investigators have reported, using change detection tasks, that PD patients, with and without dementia, do not show increased misbinding as observed in LOAD (Della Sala et al., 2012; Kozlova et al., 2020). Our results also show that the type of impairment observed in patients with LOAD is distinctly different to those observed in patients with PD, a neurodegenerative disorder which is also associated with STM deficits (Fallon, Mattiesing, Muhammed, Manohar, & Husain, 2017; Owen, Iddon, Hodges, Summers, & Robbins, 1997; Zokaei, Burnett Heyes, et al., 2014; Zokaei, McNeill, et al., 2014). Here, we used a recently developed computational model of response error for this task (Grogan et al., 2019) to demonstrate doubly dissociable underlying sources of error for LOAD compared to PD. Even without dementia, but non-significantly different disease durations, PD patients show increased guessing compared both HCs and individuals with SCI (Fig. 3b). Importantly, this deficit was observed, despite the fact that on simple indices of identification and localization performance PD patients were not significantly impaired compared to healthy controls (Fig. 2a).

That the nature of STM impairments in patients with PD is different to that in LOAD has been suggested by the results of previous investigations which used a continuous response paradigm testing colour-orientation bindings. Those studies reported that PD patients and people at risk of developing PD make significantly more random guessing responses (Rolinski et al., 2015; Zokaei, McNeill, et al., 2014). However, the performance of LOAD and PD cases has not previously been compared directly on the same continuous reproduction task, as here. It is possible that increased guessing on STM tasks in PD are manifestations of lapses in attention, resulting in an all or none memory recall (Zokaei & Husain, 2019). There is now considerable evidence of fluctuations in attention in disorders associated with Lewy body pathology, as in PD (O'Dowd et al., 2019). It is also possible that visuospatial processing deficits in patients, independent of any impairments in attentional fluctuations, might be a contributing factor to increased random response. Future research might profitably focus on understanding the link between attentional or visuospatial deficits and the type of STM impairment observed in PD.

In this study, we further explored the selectivity of STM deficits on our task by comparing performance in patients with LOAD to a group of individuals with SCI. Patients with SCI express deficits in cognition, but do not demonstrate any clinical symptoms at the time of testing. However, recent studies have shown that SCI represents a heterogenous group of individuals, many with psychiatric disorders such as depression, anxiety or mood disorders but a few at risk of developing dementia in the longer-term (Amariglio et al., 2012; Buckley et al., 2013; Hohman, Beason-Held, & Resnick, 2011; Mitchell, Beaumont, Ferguson, Yadegarfar, & Stubbs, 2014; Slavin et al., 2010; Stogmann et al., 2016). This group who present to the clinic with memory concerns provides an interesting control to test the selectivity of STM impairments we observed. Interestingly, in the present study, compared to LOAD or PD, SCI patients *overall* did not demonstrate any impairment in short-term retention of object-location bindings. Thus, as a group, they do not show the pattern of misbinding that we have observed in LOAD here and in pre-symptomatic FAD (Liang et al., 2016). Nevertheless, this task might be useful to detect and track longitudinally ‘outliers’ who show abnormally high misbinding at presentation, despite performing normally on standard cognitive screening.

Evidence in favour of pursuing this possibility comes from a study (Koppara et al., 2015) that assessed visual STM in patients with subjective cognitive decline (SCD) using the same change detection task developed by Parra and colleagues (Della Sala et al., 2012; Guazzo et al., 2020; Parra et al., 2009, 2010, 2011, 2015). Unlike our findings, that investigation reported that SCD cases showed increased misbinding compared to healthy controls, but not as high as patients with mild cognitive impairment (MCI). It is possible that the sample of SCD cases in that study might be different to the SCI group in our study. It is now widely acknowledged that a very small proportion of such cases will go on to develop dementia, but most will not (Howard, 2020). The overall findings in any group might therefore depend upon the percentage who are in the earliest preclinical phases of AD, and that proportion might be relatively small in our sample, while it might have been larger in the study of Koppara and colleagues. Long-term follow up of cases is therefore crucial to establish whether increased visual STM misbinding in any one individual is an important early cognitive marker of preclinical AD, in the context of patients who present to memory clinics and are suspected to have an underlying neurodegenerative condition, even though cognitive screening does not reveal significant deficits.

Together, our findings provide support for the selective impairment in short-term retention of bound features in patients with LOAD that is distinct to those observed in healthy controls, the SCI group we studies and patients with PD who, even without dementia, demonstrated a separate, distinctively different pattern of STM impairment. The task used here provides a relatively rapid means to measure STM and sources of error in performance. It has the potential to inform clinical practice and assessment.

## Author contribution

Conceptualization: NZ, SGM and MH; Data curation: NZ, AS, AK, DD, OP AND ES; Formal analysis: NZ and SGM; Funding acquisition: NZ and MH; Investigation: all authors; Methodology: NZ, SGM and MH; Project administration: NZ; Roles/Writing-original draft: NZ and MH; Writing-review & editing: all authors.
